# Molecular Biomarkers and More Efficient Therapies for Sepsis

**DOI:** 10.3390/biomedicines13020468

**Published:** 2025-02-14

**Authors:** Wen-Lin Su, Sheng-Kang Chiu, Chih-Hao Shen, Yi-Ting Chen

**Affiliations:** 1 Division of Pulmonary and Critical Care Medicine, Taipei Tzu Chi Hospital, Buddhist Tzu Chi Medical Foundation, No. 289, Jianguo Rd., Xindian Dist., New Taipei City 23142, Taiwan; 2 School of Medicine, Tzu Chi University, No. 701, Sec. 3, Chung Yang Rd., Hualien City 970, Taiwan; csk33kimo@hotmail.com (S.-K.C.); kateytc@gmail.com (Y.-T.C.); 3 Division of Infectious Diseases, Department of Internal Medicine, Taipei Tzu Chi Hospital, Buddhist Tzu Chi Medical Foundation. No. 289, Jianguo Rd., Xindian Dist., New Taipei City 23142, Taiwan; 4 Division of Pulmonary and Critical Care Medicine, Department of Internal Medicine, Tri-Service General Hospital, National Defense Medical Center No. 325, Sec. 2, Cheng-Kung Road, Neihu 114, Taipei 11490, Taiwan; potato652@yahoo.com.tw; 5 Department of Critical Care Medicine, Hualien Tzu Chi Hospital, Buddhist Tzu Chi Medical Foundation, No. 707, Sec. 3, Chung Yang Rd., Hualien City 970, Taiwan

## 1. Introduction

**Figure 1:**
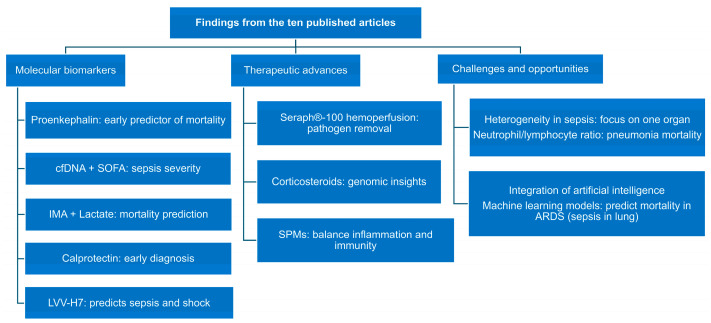
This updated figure exclusively reflects the findings derived from the ten published articles. The insights are categorized as molecular biomarkers and therapeutic advances.

Sepsis remains a leading cause of morbidity and mortality worldwide, representing a substantial burden on healthcare systems [1,2,3]. Furthermore, sepsis is an ill-defined syndrome with an immunopathophysiology characterized by simultaneous hyperinflammation and immune suppression, which makes it challenging to address varying immune statuses at different stages of sepsis [4]. Nanotechnology represents a promising trend in the future diagnosis and management of sepsis [5]. Recent advancements in molecular biomarkers and innovative therapeutic strategies offer promising avenues for addressing the multifaceted challenges of sepsis. This editorial synthesizes the key findings from recent studies (Figure 1), providing a roadmap for future research and clinical applications.

## 2. Advances in Molecular Biomarkers

### 2.1. Proenkephalin as an Early Predictor of Mortality

Emerging biomarkers such as proenkephalin (PENK) have shown potential in predicting in-hospital mortality among septic shock patients, demonstrating strong correlations with metabolic and inflammatory biomarkers [6]. PENK’s utility underscores the importance of integrating point-of-care diagnostics in early sepsis management.

### 2.2. Cell-Free DNA (cfDNA) Combined with SOFA in Severity Assessment

cfDNA, including nuclear and mitochondrial components, has been identified as a marker for sepsis severity [7]. Its combination with clinical scores such as SOFA enhances diagnostic precision and prognostication. This underscores the potential of multiplex biomarker panels in stratifying sepsis risk.

### 2.3. Ischemia-Modified Albumin (IMA) and Lactate in Mortality Prediction

The combination of IMA and lactate levels provides a robust tool for predicting mortality in septic shock patients [8]. This synergistic approach exemplifies how biomarkers can complement each other for enhanced predictive accuracy.

### 2.4. Calprotectin in Early Diagnosis of Infections

Calprotectin’s ability to predict bacterial infections offers a cost-effective solution for early sepsis detection, reducing ICU stays and mortality [9]. This health economic analysis highlights calprotectin’s economic and clinical benefits as part of early intervention strategies.

### 2.5. LVV-Hemorphin-7 (LVV-H7) in Predicting Sepsis

LVV-H7, a metabolite of cell-free hemoglobin catalyzed by cathepsins D and G during infection, shows potential for predicting sepsis and shock in critically ill patients with acute changes in SOFA scores [10]. These results highlight the utility of cell-free hemoglobin metabolites in sepsis prognostication.

## 3. Innovative Therapeutic Approaches

### 3.1. Seraph^®^-100 Hemoperfusion

Novel therapies such as the Seraph^®^-100 hemoperfusion device have demonstrated efficacy in removing bacterial pathogens in a simulated hemoperfusion study, representing a breakthrough in extracorporeal therapies for sepsis-related bacteremia [11].

### 3.2. Corticosteroids and Genomic Insights

Corticosteroids, long debated for their role in sepsis management, may benefit from genomic and transcriptomic stratification to identify responsive subgroups [12]. These therapies modulate the immune response, stabilize the cardiovascular system, and potentially facilitate organ restoration. This personalized approach could optimize outcomes while minimizing risks.

### 3.3. Specialized Pro-Resolving Mediators (SPMs)

In sepsis, severe inflammation occurs early, followed by paradoxical immunosuppression in later stages. SPMs offer a dual advantage by resolving inflammation without inducing immunosuppression [13]. Their role in managing the immunosuppressive phase of sepsis provides a balanced therapeutic strategy.

## 4. Challenges and Opportunities

### 4.1. Heterogeneity in Sepsis

Sepsis is not a singular disease but a syndrome with diverse etiologies and manifestations. Biomarker-driven phenotyping can aid in tailoring therapies to individual patient profiles, addressing this heterogeneity. Pneumonia, a major cause of sepsis, may benefit from predictors such as the neutrophil/lymphocyte ratio and pneumonia severity index for mortality risk assessment [14].

### 4.2. Integration of Artificial Intelligence

Machine learning and imaging algorithms are emerging as powerful tools for sepsis diagnosis and monitoring. These technologies can complement molecular biomarkers to refine risk stratification and therapeutic decisions. For example, integrating P/F ratios and chest X-ray data in machine learning models may help predict mortality in SARS-CoV-2-associated ARDS [15].

## 5. Future Directions

**Figure 2:**
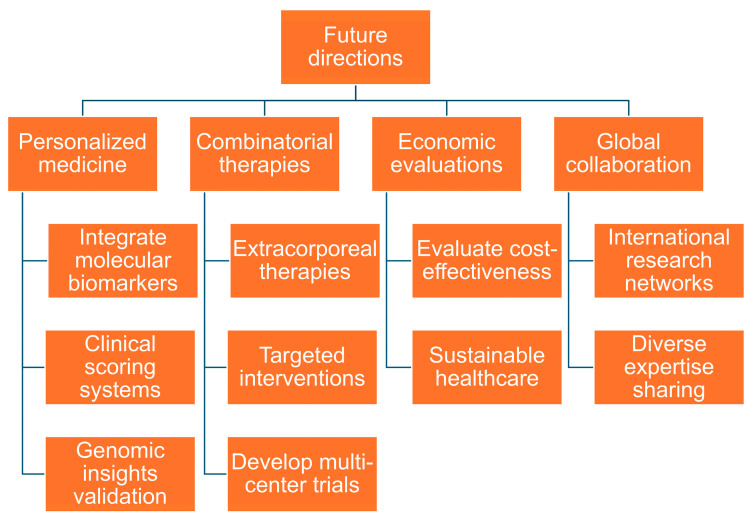
This figure illustrates the future directions in sepsis biomarkers and therapies. It highlights key themes—Personalized Medicine, Combinatorial Therapies, Economic Evaluations, and Global Collaboration—along with their associated strategies.

As illustrated in Figure 2, the future directions focus on personalized medicine and combinatorial therapies for heterogeneous sepsis, including variations in early versus late sepsis and different infection sources. Economic evaluation and global collaboration should also be prioritized, driven by artificial intelligence to tackle this complex disease.

### 5.1. Personalized Medicine

The integration of clinical scoring systems [16] combined with molecular biomarkers [17] such as proteomics [18] and genomic insights [19] paves the way for personalized sepsis management. The different pathogens and their drug resistance and virulence may have different outcomes, such as mortality [20]. In addition, the varying definitions of infection in critically ill populations [21] and the definition of sepsis may also offer important predictions of mortality outcomes [22]. Future research should focus on validating biomarker panels in diverse populations.

### 5.2. Combinatorial Therapies

The sepsis treatment guidelines, as established by the Surviving Sepsis Campaign in 2021 [23], still lack strong evidence for molecular therapies. Combining extracorporeal therapies [24,25,26,27], targeted pharmacological interventions [28,29], and supportive care [30,31] can maximize treatment efficacy. As many multi-center trials have begun of targeted pharmacological therapy in sepsis [32,33], additional multi-center trials are needed to establish best practices for such combinatorial approaches.

### 5.3. Economic Evaluations

Given the financial burden of sepsis, cost-effectiveness analyses should be integral to the development and deployment of new biomarkers and therapies [34]. This ensures the sustainability of healthcare interventions.

### 5.4. Global Collaboration

Sepsis is a global health challenge. International collaborations can accelerate the discovery and implementation of effective solutions, leveraging diverse expertise and resources [35].

## 6. Conclusions

The convergence of molecular biomarkers, innovative therapies, and advanced technologies heralds a new era in sepsis management. By addressing the complexities of this syndrome through personalized and evidence-based approaches, we can improve outcomes for millions of patients worldwide.
